# Low-temperature large-area fabrication of ZnO nanowires on flexible plastic substrates by solution-processible metal-seeded hydrothermal growth

**DOI:** 10.1186/s40580-020-00235-6

**Published:** 2020-07-13

**Authors:** Kangeun Yoo, Wonseok Lee, Kyungnam Kang, Inhwan Kim, Daehun Kang, Dong Kyo Oh, Min Cheol Kim, Hyunsik Choi, Kwangjun Kim, Minwook Kim, Jeong Dae Kim, Inkyu Park, Jong G. Ok

**Affiliations:** 1 Department of Mechanical and Automotive Engineering, Seoul National University of Science and Technology, Seoul, 01811 Republic of Korea; 2 Department of Mechanical Engineering, Korea Advanced Institute of Science and Technology (KAIST), Daejeon, 34141 Republic of Korea; 3 Research Center for Electrical and Information Technology, Seoul National University of Science and Technology, Seoul, 01811 Republic of Korea; 4 Present Address: Technology Business Team, UT Aim Co., Ltd., Tokyo, 141-0022 Japan; 5 Present Address: Technical & Material Team, CNJ Inc., Auburn, AL 36832 USA; 6 Present Address: Etch Team, SEMES Co., Ltd., Cheonan, Chungcheongnamdo 31040 Republic of Korea

**Keywords:** Zinc oxide nanowire, Hydrothermal process, Low temperature, Metal seed, Flexible plastic, Solution process, Transparent UV sensor

## Abstract

We have developed the low-temperature conformal ZnO nanowire fabrication on flexible plastic substrates by utilizing the solution-processible metal seed-assisted hydrothermal ZnO crystallization. Structural evolution of ZnO nanowires controlled by major parameters involving growth temperature, growth time, and seed coating condition, has been systematically investigated towards uniform and large-area growth of conformal ZnO nanowires. Direct ZnO nanowire growth on flexible plastics without undergoing the high-temperature seed sintering has been realized by developing the low-temperature Ag-seeded hydrothermal ZnO nanowire growth. The nanoporous Ag layer favorable for ZnO crystal nucleation and continued nanowire growth can be reduced from the Ag ion solution coating at the temperature as low as 130 °C. This tactfully enables the selective hydrothermal growth of ZnO nanowires on the Ag patterns on flexible plastics. Such an all-solution-processible low-temperature fabrication protocol may provide an essential and practical solution to develop many diverse applications including wearable and transparent electronics, sensors, and photocatalytic devices. As one example, we demonstrate that a transparent UV sensor can be devised based on the ZNW growth on the Ag micromesh electrode.

## Introduction

Possessing wide band gap (~ 3.37 eV), large exciton binding energy (~ 60 meV), non-central symmetric wurtzite crystal structure (a = 0.3249 nm, c = 0.5205 nm), and biocompatibility, zinc oxide (ZnO) has been one of the most versatile metal oxide materials [1, 2]. ZnO and its micro- and nanostructures have accordingly enabled many diverse functional devices covering electronics, photonics, transducers, bioengineering, and so on [3–8]. While a variety of nanoscale configurations are available for ZnO, one-dimensional (1D) nanowires (NWs) may be particularly useful and available for most of the practical applications because of their structural simplicity and growth controllability [2, 9–11]. High-quality ZnO NWs (ZNWs) can be grown by the chemical vapor deposition (CVD) processes based on the metal-catalyzed vapor–liquid-solid (VLS) mechanism and/or catalyst-free vapor–solid mechanism [12–15].

Such a CVD-based route, however, typically accompanies high-temperature vacuum-chamber processing, which often limits the applicable substrate and processible area. To meet the emerging needs for flexible and scalable device fabrication, for instance on large-area plastics, the hydrothermal crystallization of ZnO nanostructures through a series of low-temperature chemical reactions in an aqueous solution bath can be suitably utilized [11, 16]. In the hydrothermal process, the resulting ZnO nanostructure morphology can be altered by the main growth parameters including bath temperature, growth time, and seed coating condition [17–22]. More critically, the seed layer typically necessary for ZnO crystal nucleation (*e.g.* textured ZnO layer) usually demands high-temperature sintering (at ~ 350 °C) of a Zn-salt solution coating [23, 24]. A reliable hydrothermal ZNW growth thus requests parametric control to obtain uniform and conformal NW morphology as well as low-temperature seed formation to allow flexible plastic substrate use.

In this work, we first investigate the evolution of ZnO nanostructures depending on the main hydrothermal growth parameters, which leads to the high-density conformal ZNW growth over a wafer-scale large area. Then we demonstrate that the nanoporous metal (*e.g.* Ag) layer reduced from the ionic solution coating at much lower temperature (< 130 °C) can work as an alternative seed for hydrothermal ZNW growth. This realizes direct ZNW growth on the common plastic substrates without resort to high-temperature seed sintering. Such a metal-seeded low-temperature ZNW growth allows scalable fabrication of flexible ZNW-based electronic devices by the selective ZNW growth on the specifically designed metal patterns. We show one example that ZNWs are selectively grown on the metal micromesh pattern, which can be directly applied for transparent UV sensors.

## Experimental

**Fig. 1:**
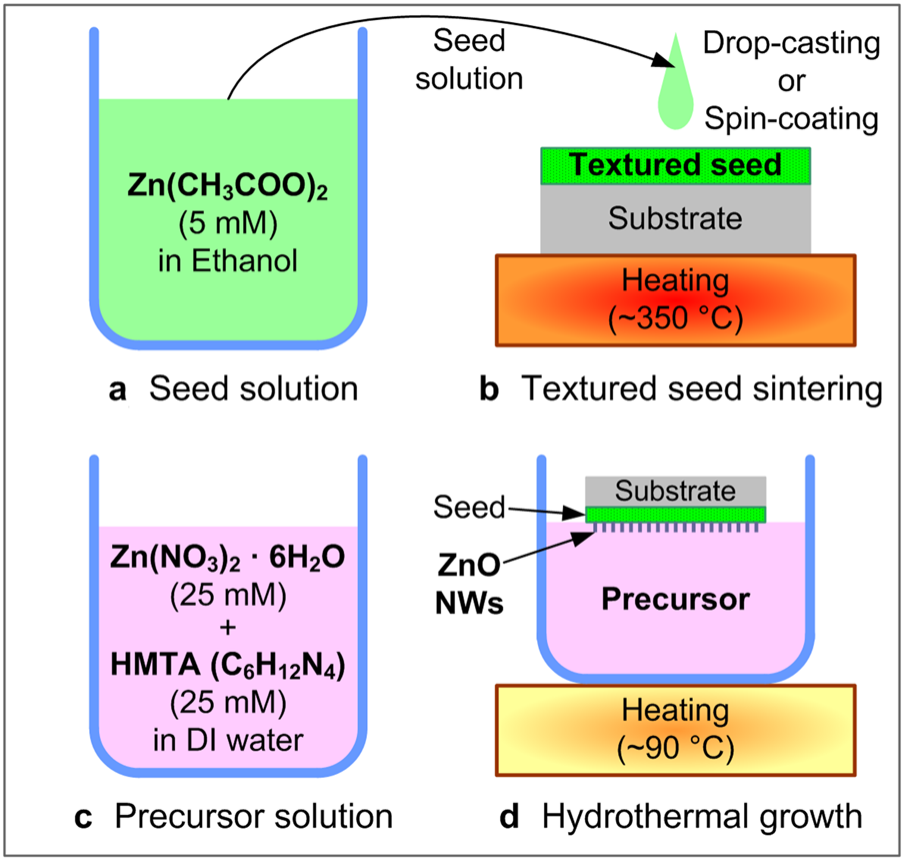
Schematic illustration of ZNW growth procedure based on hydrothermal reactions

The baseline recipes and list of reactions of hydrothermal growth of ZnO nanostructures using textured ZnO seeds have been presented in many previous reports [19–22]. Here we demonstrate one typical process used in this study. Figure 1 illustrates the overall growth procedure. First, a seed solution was prepared by dissolving zinc acetate dihydrate (Zn(CH_3_COO)_2_, reagent grade, Sigma-Aldrich Corp.) in ethanol (99.9%, Samchun Pure Chemical Co., Ltd.) with the concentration of 5 mM (Fig. 1a). The seed solution was drop-cast or spin-coated (1000 rpm, 1 min) on the target substrate (*i.e.* typically Si unless otherwise specified; glass, polyethylene terephthalate (PET), and polycarbonate (PC) for some cases (see Fig. 6); pre-treated by O_2_ plasma to enhance the coating quality, as needed). Then the substrate was dried naturally or under slight heating (*e.g.* by putting on a 70 °C-heated hot plate) inside a fume hood. This seed solution coating and drying was repeated five times. The seed solution-coated substrate was put on a 350 °C-heated hot plate for 20 min for the sintering of the textured ZnO seed (Fig. 1b). A precursor solution was prepared by adding zinc nitrate hexahydrate (Zn(NO_3_)_2_·6H_2_O, reagent grade, 98%, Sigma-Aldrich Corp.) and hexamethylenetetramine (HMTA; C_6_H_12_N_4_, ACS reagent, ≥ 99.9%, Sigma-Aldrich Corp.) in deionized (DI) water with both the concentrations of 25 mM and stirring for 1 h at 140 °C (Fig. 1c). Before growth, the precursor solution was typically vacuum-filtered by using a membrane filter (pore size 0.1 µm) to remove undissolved impurities. The seed-formed substrate was carefully floated on the precursor solution, with the seed-coated side facing down. For growth, the bath heating was maintained by using a hot plate or heating mantle at the controlled temperature (60–120 °C, typically 90 °C) for a controlled amount of time (45–210 min, typically 150 min) (Fig. 1d). After growth was done, the sample was cooled down, delicately washed by DI water, and dried by slight nitrogen blowing.

For the metal-seeded ZNW growth, the isopropyl alcohol (IPA)-based Ag-ion solution ink (TEC-CO-011, InkTec Co., Ltd.; ‘Ag ink’ hereafter) was diluted in IPA with 1:1 volume ratio by sonication. This Ag ink was spin-coated onto the target substrate (*i.e.* glass, PC, and polyimide (PI)) typically at 2000 rpm for 20 s. After soft-baking at 90 °C for 1 min, the substrate was annealed at 130 °C for 5 min to reduce Ag ions to form the nanoporous Ag metal thin film. ZNW growth was conducted on this substrate by following the same procedure described in Fig. 1c, d. The growth time was controlled to be typically longer for the Ag-seeded cases (6–12 h, typically 9 h) to ensure reliable ZNW growth; this difference in growth rate is under subsequent research. For crystal structure characterization, a Bruker DE/D8 Advance X-ray Diffraction (XRD) spectroscopy (X-ray wavelength of 0.154 nm) was used for the 2 theta range of 20°–80°. For the Ag micromesh fabrication, a UV photography process was applied on the 2 µm-thick negative photoresist (DNR-L300-40 (120 cP), Dongjin Semichem Co., Ltd.)-coated Ag film by using a Karl Suss MA8 instrument, followed by the PR develop (AZ 300 MIF developer, Merck & Co., Inc.), metal wet etching (Aluminum etchant type A, Transene Co., Inc.), and PR strip (acetone and IPA) processes. The typical linewidth/spacing geometry of the micromesh pattern was 50/250 µm. All scanning electron microscopy (SEM) images were taken by using a field-emission SEM instrument (JSM-6700F, JEOL Ltd.), typically operated at 10 kV. For UV sensing characterization for the devices comprising ZNWs grown on the Ag micromesh electrodes, a custom-built 4-point probe station and a UV source (LAX-C100, Asahi Spectra USA Inc.) were used at ambient environment. The photocurrent generated upon UV illumination with controlled power density was measured at the applied bias of 0.1 V.

## Results and discussion

### Structural evolution of ZNWs by controlling hydrothermal growth parameters

#### Effect of growth temperature

**Fig. 2:**
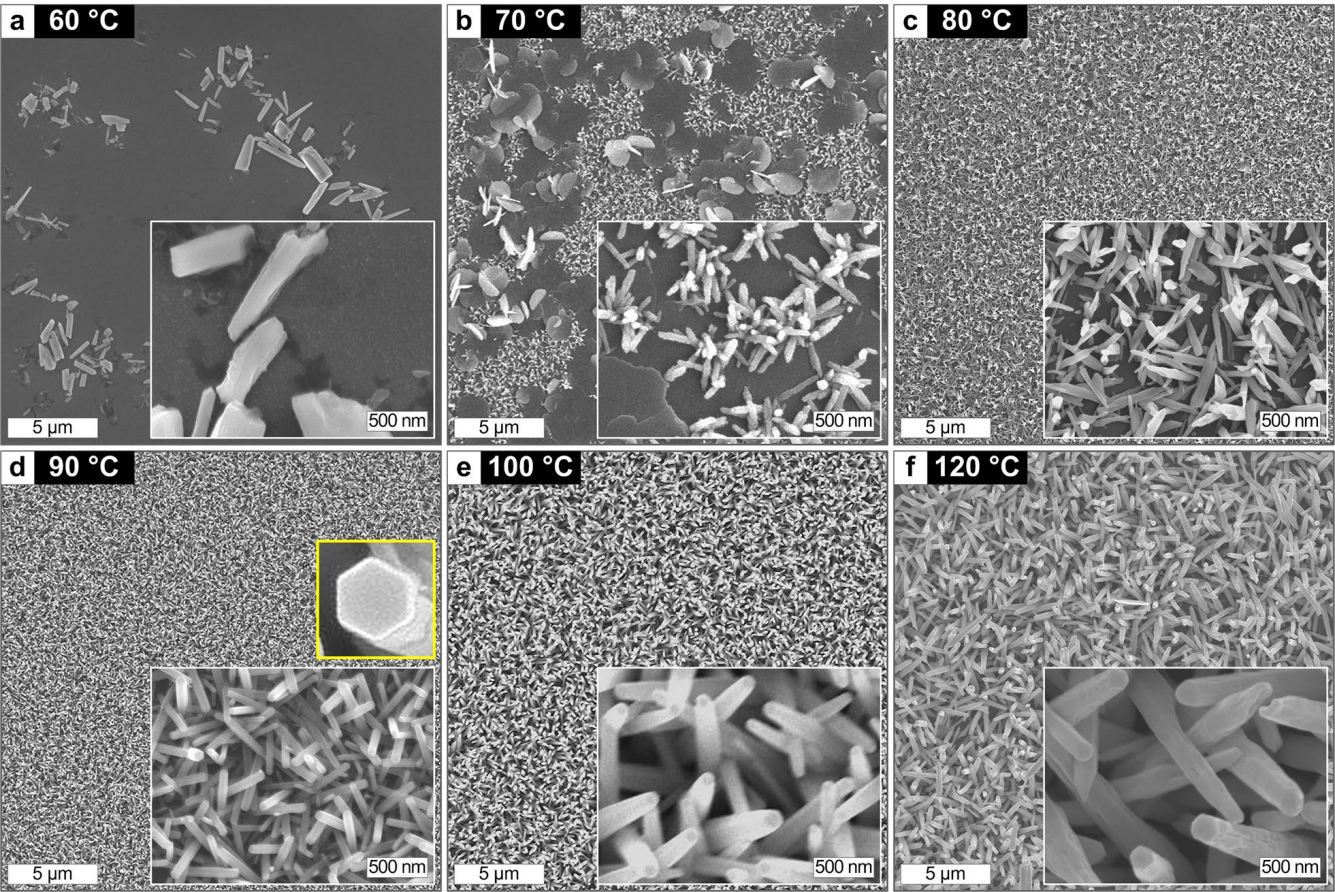
SEM images of the ZNWs grown by the hydrothermal process at different temperatures. Growth time is fixed to 150 min for all cases. Insets show enlarged views of ZNW morphologies

Figure 2 shows the results of hydrothermal ZNW growths at different temperatures with the growth time of 150 min and other conditions fixed. ZnO nanostructures are not fully developed at lower temperatures (60–70 °C; Fig. 2a, b), and begin to evolve to 1D structure as the temperature increases (80 °C; Fig. 2c). At the optimal temperature in our experimental space (90 °C, Fig. 2d), high-density ZNWs are grown in a uniform and conformal fashion with the diameter of ~ 60–80 nm. The inset to Fig. 2d clearly discloses the hexagonal section growing along the energetically favorable *c* axis (*i.e.* [0001]) for the wurtzite ZnO crystal [1]. As the growth temperature further increases, the ZNW morphology overall bulks up with the diameter larger than 100 nm while the density of ZNWs decreases (100–120 °C; Fig. 2e, f). The nucleation of ZnO crystals towards ZNW array is generally facilitated as the temperature increases in the thermodynamically driven hydrothermal ZNW growth [18, 21, 25]. Non-NW morphology such as plates and flowers is often obtained at lower temperature [26], while ZNWs become plump with decreased density when the temperature is too high [27]. Here we choose 90 °C as our standard growth temperature which leads to high-density, conformal ZNW arrays compared to the other temperatures growths in our experimental space.

#### Effect of growth time

**Fig. 3:**
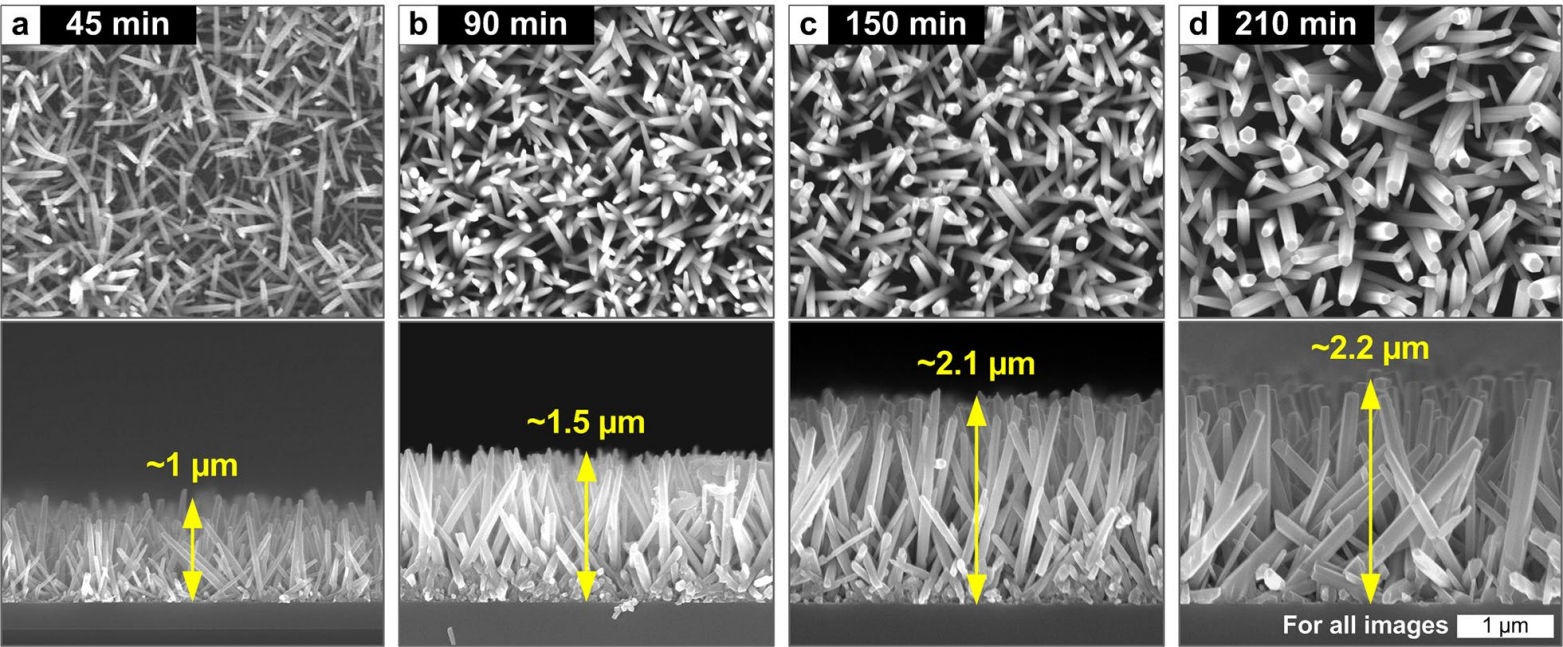
SEM images of the ZNWs grown by the hydrothermal process for different growth times. Growth temperature is fixed to 90 °C for all cases

Growth time is another important parameter controlling the ZNW structures, especially the length of ZNWs [21, 27], in the hydrothermal growth process. Figure 3 shows the top and side views of ZNWs grown for different growth times at 90 °C, indicating that ZNWs are generally grown longer at increased growth time. Notably, as the growth time extends, the growth rate gradually decreases (*i.e.* 22.2 nm/min, 16.7 nm/min, 14.0 nm/min, and 10.5 nm/min for the growths for 45, 90, 150, and 210 min, respectively) while the average diameter apparently increases. This suggests that the axial growth becomes saturated while radial growth progresses in our experimental condition. The axial and radial growth modes of ZNWs may collectively depend on the pH, composition, and concentration of the precursor solution as well as the diffusion dynamics on the ZNW surfaces [27–32], which should be an interesting subject for future analysis.

#### Effect of seed coating condition and uniform large-area ZNW growth

**Fig. 4:**
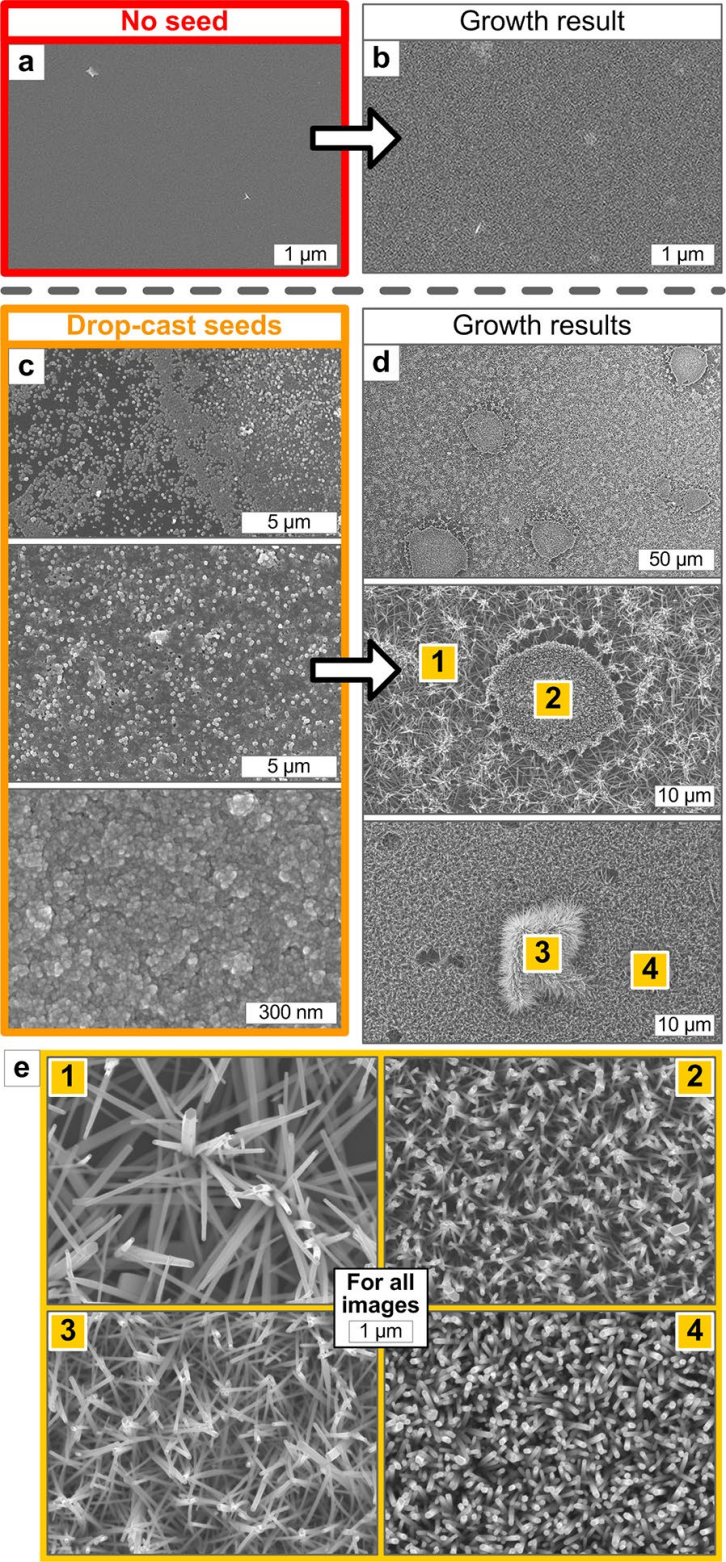
SEM images showing hydrothermal ZNW growth results using (**a**, **b**) no seed and (**c**–**e**) drop-cast seed coatings. Enlarged SEM images (1–4) shown in **e** are taken from four different spots marked in (d). All growths are done at 90 °C for 150 min

The seed coating condition is critical for conformal and uniform ZNW growth from initial ZnO crystal nucleation [17, 20, 22, 25, 26]. After briefly confirming that ZNW growth is not successful with no seed coating (Fig. 4a, b) [17], we first examine the growth result from the drop-cast seed coating (Fig. 4c–e). Figure 4c shows the SEM images of the drop-cast and sintered ZnO seed surfaces taken from three different spots on a 2 × 2 cm sized Si piece, revealing the poor uniformity and uneven grains of the seed layer due to the non-uniform spreading and sintering of seed solution drops. The ZNWs grown on such a drop-cast seed (at 90 °C for 150 min) consequently show heterogeneous morphologies as shown in Fig. 4d, e.

**Fig. 5:**
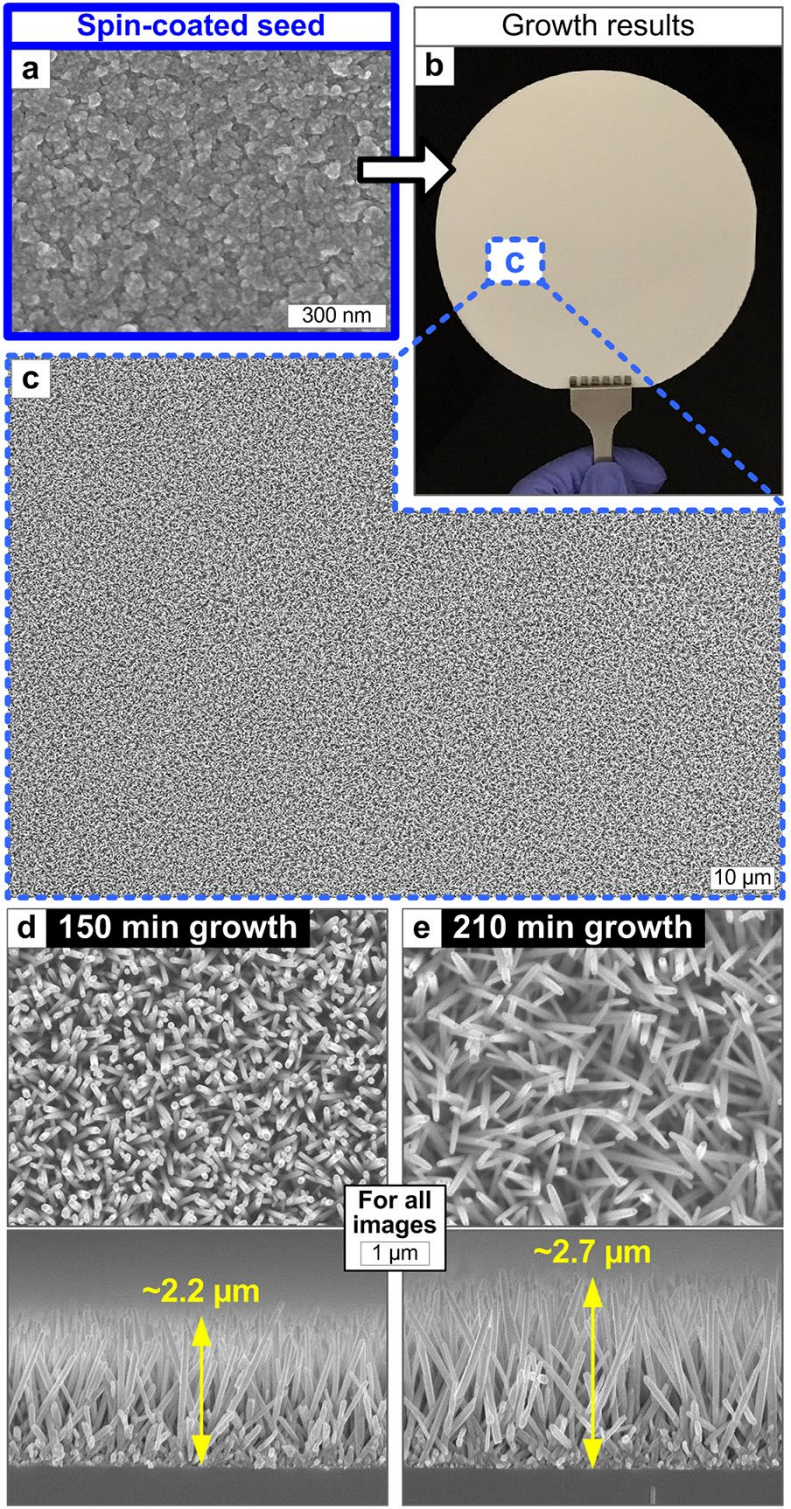
Hydrothermal ZNW growth results using spin-coated seed coatings: **a** SEM image of the nanoscale texture of the seed surface, **b** optical image and **c** SEM image of the 4” wafer-scale ZNW growth result, and **d**–**e** top and side morphologies of the ZNWs grown on spin-coated seed coatings for different growth times. All growths are done at 90 °C

Spin-coating of the seed solution can improve the uniformity and scalability of ZNW growth [20–22]. Figure 5a shows the spin-coated and sintered ZnO seed on a 4” Si wafer; not shown here, it is noteworthy that almost identical nanoscale seed textures were observed at nine different spots. The ZNWs grown on this wafer-scale seed layer exhibit excellent uniformity (Fig. 5b, c). The ZNW morphology depending on growth time indicates the similar trend as demonstrated in Fig. 3; a slight increase in growth rate may be possibly due to the enhanced hydrothermal reactions over a smooth and homogeneous seed surface without encountering unfavorable spots such as blanks and lumps.

### Low-temperature ZNW growth on flexible plastics by using solution-processible metallic seeds

#### Incompatibility of textured ZnO seeds with flexible plastics

**Fig. 6:**
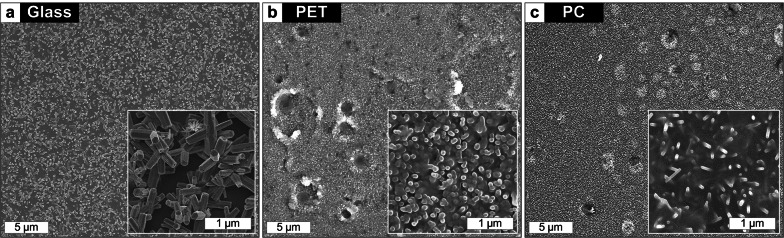
SEM images of the ZNW growth results on glass and plastic substrates after coating and annealing the Zn(CH_3_COO)_2_ solution-based seed at 70 °C (for PET) and 90 °C (for glass and PC). All growths are done at 90 °C for 150 min

While the ZnO seed layer fabricated by the spin-coating of a Zn(CH_3_COO)_2_-based solution realizes a wafer-scale large-area ZNW growth in a conformal and uniform fashion, the reliable textured ZnO seed formation still demands the thermal decomposition of Zn(CH_3_COO)_2_ at the temperature as high as 350 °C [23, 24, 33]. Such an indispensable high-temperature sintering obstructs the use of flexible plastics as the substrates for faithful ZNW growth. Attempts to grow ZNWs on the glass and common plastics (PET and PC) at 90 °C after Zn(CH_3_COO)_2_ solution coating and annealing at 70–90 °C at which the plastics can anyhow sustain, end up with non-uniform and undeveloped ZnO nanostructures (Fig. 6) due to unfulfilled seed supports. An alternative seed preparation technique which needs not high-temperature sintering is thus called for to move towards direct growth of ZNWs on flexible plastic substrates.

#### Low-temperature ZNW growth by using solution-processible Ag layers as alternative seeds

Hydrothermal ZNW growths based on Zn-based seeds but without involving high-temperature sintering have recently been demonstrated in various ways ranging from physical vapor deposition of Zn/ZnO layer [25, 34] to ZnO-containing colloid coating [28, 35, 36]. While all these methods are effective for growing ZNWs on plastics, application to practical flexible devices may require additional steps for electrically connecting ZNWs to the device circuits. One attractive approach to this end can be the selective direct growth of ZNWs on the conductive surfaces such as metals [37–39] and graphene [40, 41] that are favorable for hydrothermal crystal growth. Preparing the metal layers without relying on vacuum deposition may further increase the process throughput with simplified apparatus and reduced cost. Namely, forming the metal seed layer via the reduction of a metal-ion solution coating and growing ZNWs via low-temperature hydrothermal process on a flexible plastic will realize ‘all-solution-processible’ device fabrication throughout the entire procedure.

**Fig. 7:**
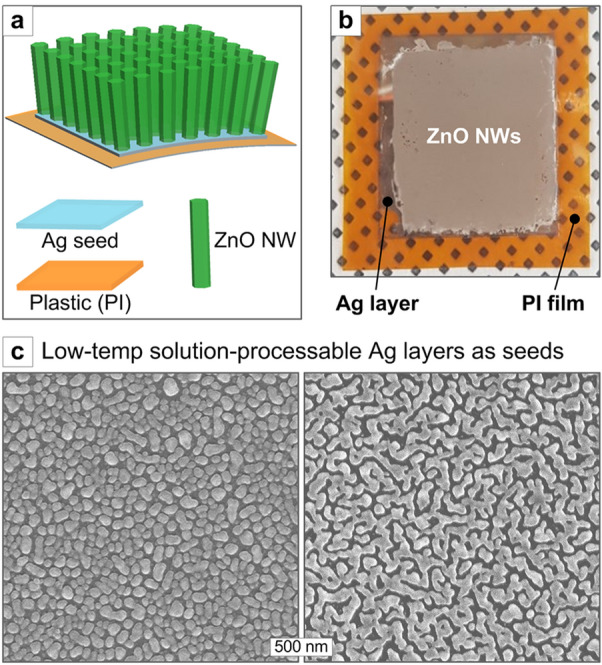
**a** Schematic drawing of the Ag-seeded ZNW growth on the plastic substrate, **b** optical image of ZNWs grown on the Ag-coated PI film, and **c** SEM images of Ag seed layers reduced from the Ag ink solution coatings (nanoporous morphology tuned by controlling Ag ink coating condition)

**Fig. 8:**
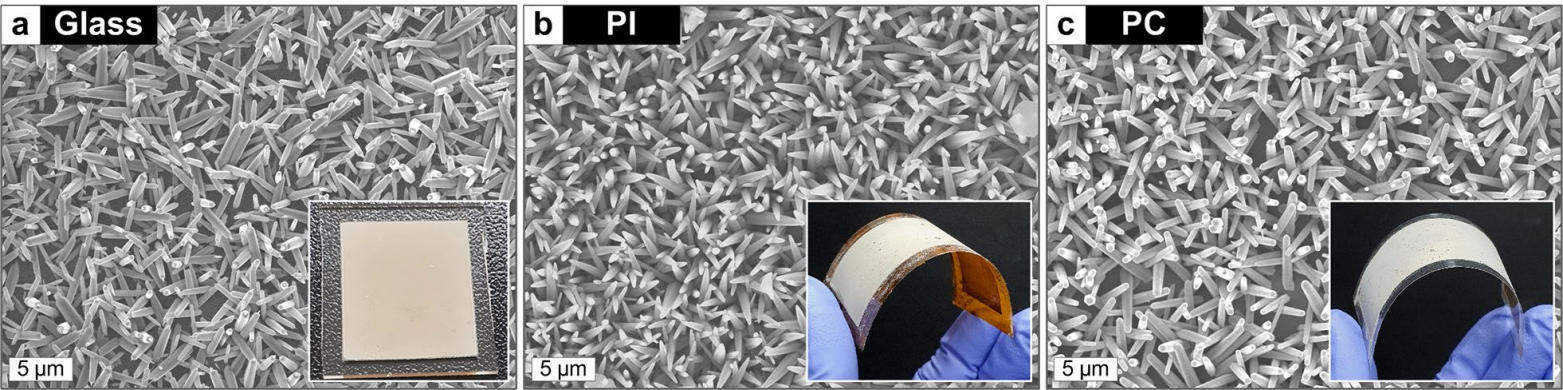
SEM images of ZNWs grown on the Ag-seeded surfaces: **a** glass, **b** PI, and **c** PC. Insets show the optical images of the fabricated samples. All growths are done at 90 °C for 9 h

Figure 7a, b depicts such a tactful procedure; the Ag ink solution (see *Experimental*) is first coated on a flexible plastic and then is reduced to the nanoporous Ag layer (Fig. 7c) at the temperature as low as 130 °C, and ZNWs are selectively grown on the Ag surface. The morphology of Ag layers, regarding the thickness, porosity, and grain shape, etc., can be readily tailored by controlling the Ag ink concentration and coating speed as well as the thermal reduction condition, which is being systematically investigated for the separate work. This solution-processed Ag seed can serve as the ZNW growth seed on various substrates; Fig. 8 shows the exemplary results of conformal ZNW growths on glass, PI, and PC substrates. The underlying mechanism on the Ag-mediated hydrothermal ZNW growth is attributed to the interactions between oxidized Ag surface and ZnO complexes in the solution [42, 43]. Briefly, the ionized Ag surface generating on the native oxide (*i.e.* Ag_2_O) layer would electrostatically attract the negatively charged Zn ion complexes (*i.e.* Zn(OH)_3_^−^, Zn(OH)_4_^2−^) [16, 43] generating in the OH^−^-rich precursor solution (*i.e.* pH ~ 8–9 due to amine base HMTA), facilitating ZnO nucleation on the Ag-seeded substrate. The (natively oxidized) Ag surface and ZnO exhibit excellent lattice match [42, 43], which would further promotes ZnO crystal growth towards conformal and uniform ZNW structure.

#### Advanced study for the parametric controls and transparent UV sensor device designs

**Fig. 9:**
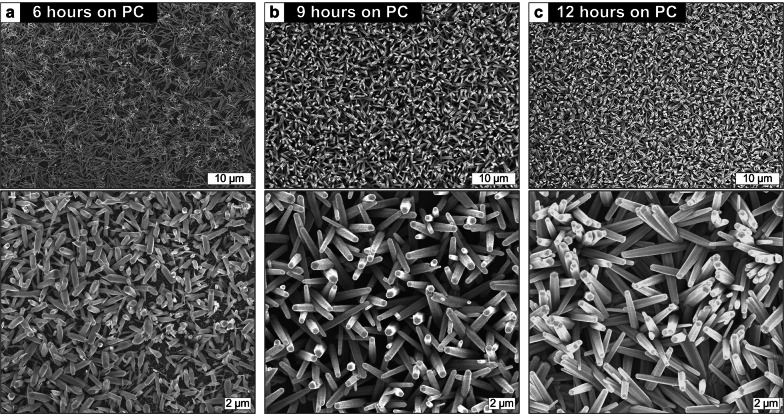
SEM images of ZNWs grown on the Ag-seeded PC substrates for different times. Top and bottom rows show low and high magnification views, respectively, for each growth time. All growths are done at 90 °C

**Fig. 10:**
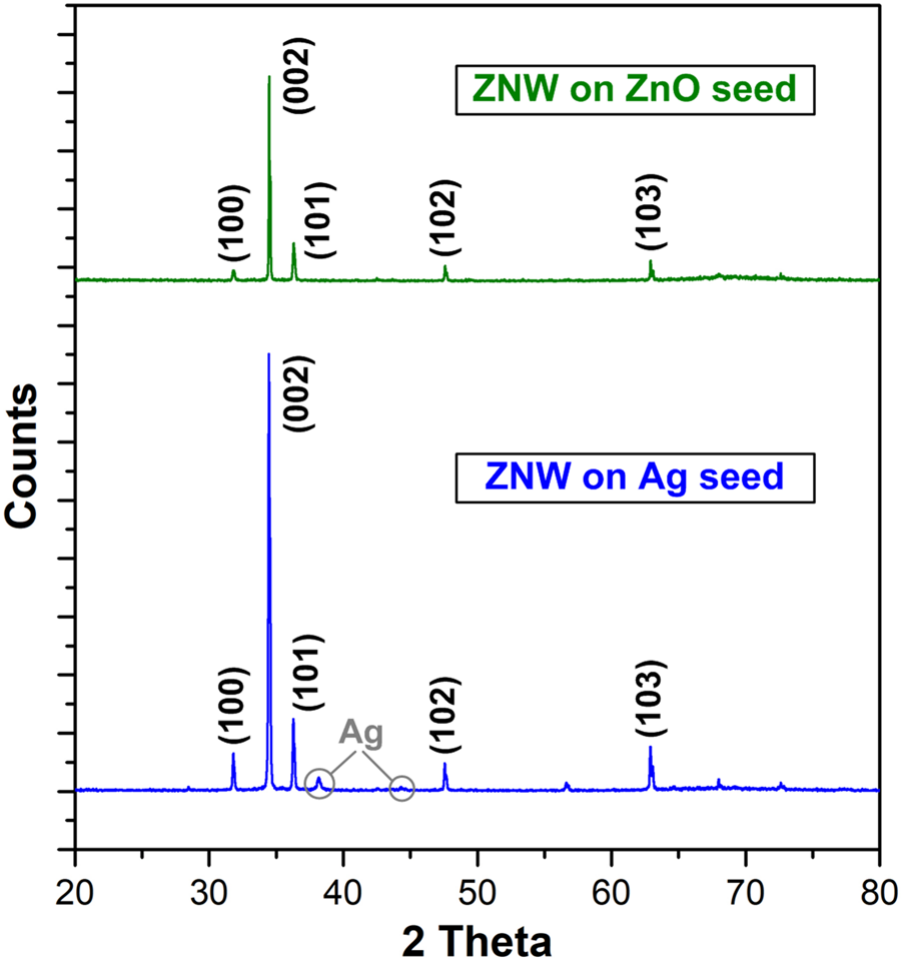
XRD spectra of ZNWs grown on the textured ZnO seed (top) and solution-processed Ag seed (bottom). All growths are done at 90 °C

The length, density, and aspect ratio of ZNWs grown by using the Ag seed can be controlled by regulating the hydrothermal growth parameters as well as the Ag seed formation conditions. While the detailed study on such parametric controls is underway, Fig. 9 demonstrates the exploratory result for ZNW growths on the Ag-seeded PC substrates for different growth times. ZNWs are fully developed from 6 to 9 h and are scaled up as the growth time extends; this trend is similar to the time series of ZnO-seeded growth (Fig. 3) yet reveals generally much slower growth rate, which awaits further investigation. The XRD spectra taken from the ZNWs grown by using the textured ZnO seed and solution-processed Ag seed comparatively plotted in Fig. 10 show very sharp narrow peaks dominantly emerging at multiples of the (001) index. This confirms that ZNWs are highly crystalline with excellent vertical alignment [44].

**Fig. 11:**
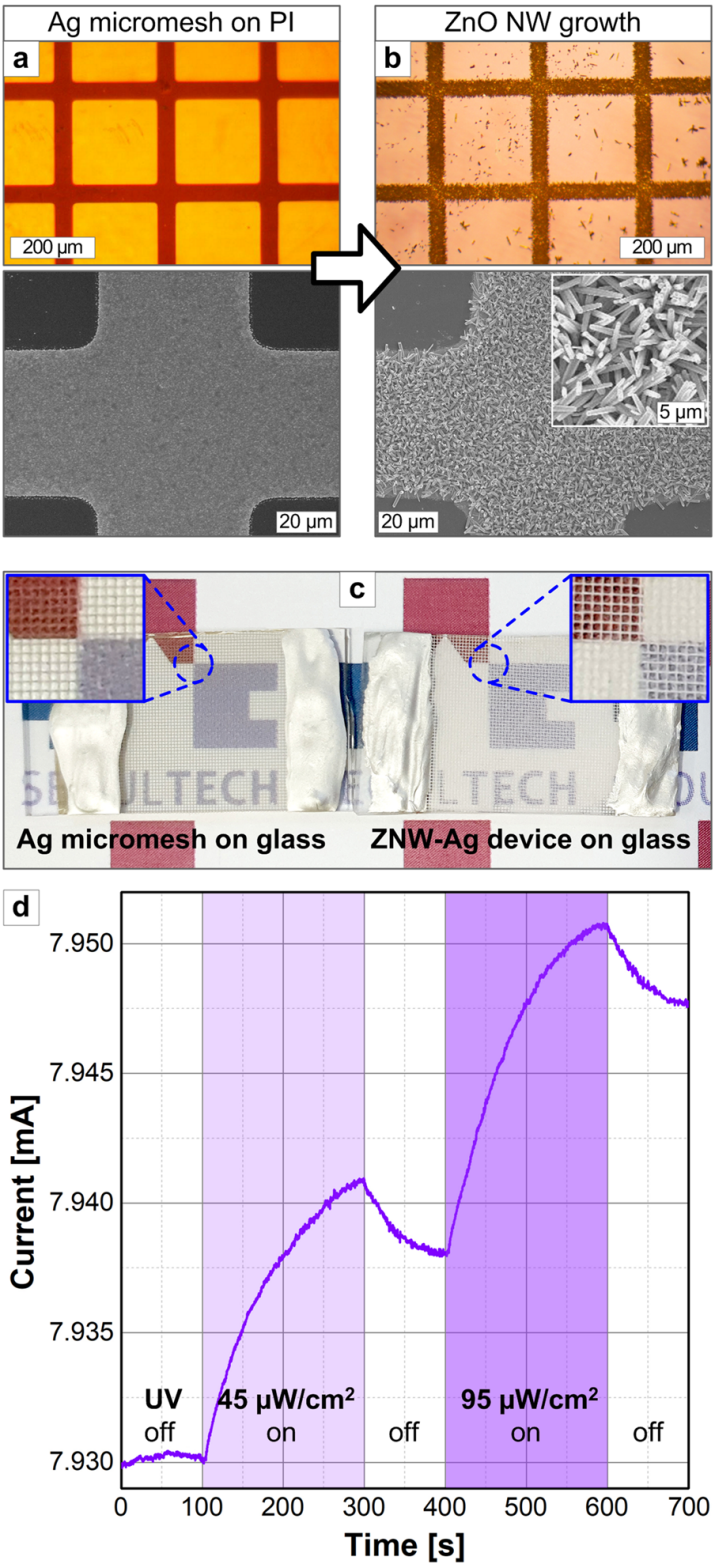
Patterned ZNW growth on the Ag micromesh pattern (linewidth/spacing = 50/250 µm) formed on a PI film: **a** before and **b** after ZNW growth. Growth is done at 90 °C for 9 h. Demonstration of the ZNW-Ag structures micropatterned on glass substrates as transparent UV sensors: **c** optical images for the sample with the 50 µm linewidth and 250 µm spacing, comparing before and after ZNW growth and **d** UV sensing characterization of the ZNW-integrated Ag micromesh system for different UV intensities

The ZNW growth selectively occurring on the Ag surface practically enables the direct fabrication of patterned ZNW structures on specifically designed Ag patterns. Figure 11 exemplifies that high-density ZNWs are selectively grown on the optically transparent Ag micromeshes photolithographically fabricated on flexible and/or transparent substrates. The observation of few ZNWs on a bare PI surface (Fig. 11b) confirms the Ag-mediated growth mechanism. Figure 11c further demonstrates that this ZNW-integrated Ag micromesh system, for instance fabricated on the glass substrate, can be utilized as the transparent UV sensor. The free electrons separated from the excitons generated inside ZnO upon UV illumination can be collected through the Ag electrodes, leading to the photocurent signal [12, 13]. As the valid sensing architecture, the photocurrent varies depending on UV power density as shown in Fig. 11d. While not fully tuned here, the sensing performance as well as device transmittance can be optimized by adjusting the ZNW-Ag structures and sensing conditions. Such a strategy may pave the way for the facile fabrication of various transparent and flexible devices capitalizing ZNWs electrically connected to conducting frameworks.

## Conclusions

In summary, we have investigated the structural evolution of hydrothermally grown ZnO nanostructures depending on the main process parameters including growth temperature and time and seed preparation. We have accordingly established the favorable condition for wafer-scale conformal ZNW growth with excellent uniformity. In order to realize the ZNW growth on flexible plastics at low temperature by circumventing the high-temperature (*i.e.* 350 °C) textured ZnO seed preparation, we have developed the Ag-seeded ZNW growth utilizing the reduction of Ag ion solution coating to Ag layer. This can be conducted at the temperature as low as 130 °C in the manner of solution processing throughout the entire process from seed preparation to hydrothermal growth. Such an all-solution ZNW growth protocol tactfully realizes the direct fabrication of transparent and flexible functional devices by selectively growing ZNWs on the metal electrode patterns. The transparent UV sensor has been exploratively demonstrated as one example. Many applications may benefit from the methodology presented in this work, including, but not limited to flexible electronics, wearable sensors, and transparent photonic and energy-conversion devices.

## Data Availability

All data generated or analyzed during this study are included in the published article.

## Abbreviations

ZnO: Zinc oxide
ZNW: Zinc oxide nanowire
CVD: Chemical vapor deposition
VLS: Vapor-liquid–solid
PET: Polyethylene terephthalate
PC: Polycarbonate
DI: deionized
HMTA: Hexamethylenetetramine
PI: Polyimide
IPA: Isopropyl alcohol
PR: Photoresist
SEM: Scanning electron microscopy
